# Ab initio thermodynamics reveals the nanocomposite structure of ferrihydrite

**DOI:** 10.1038/s42004-021-00562-7

**Published:** 2021-09-20

**Authors:** Michel Sassi, Anne M. Chaka, Kevin M. Rosso

**Affiliations:** Physical and Computational Sciences Directorate, Pacific Northwest National Laboratory, Richland, WA 99352 USA

**Keywords:** Density functional theory, Nanoparticles, Structural properties, Geochemistry

## Abstract

Ferrihydrite is a poorly crystalline iron oxyhydroxide nanomineral that serves a critical role as the most bioavailable form of ferric iron for living systems. However, its atomic structure and composition remain unclear due in part to ambiguities in interpretation of X-ray scattering results. Prevailing models so far have not considered the prospect that at the level of individual nanoparticles multiple X-ray indistinguishable phases could coexist. Using ab initio thermodynamics we show that ferrihydrite is likely a nanocomposite of distinct structure types whose distribution depends on particle size, temperature, and hydration. Nanoparticles of two contrasting single-phase ferrihydrite models of Michel and Manceau are here shown to be thermodynamically equivalent across a wide range of temperature and pressure conditions despite differences in their structural water content. Higher temperature and water pressure favor the formation of the former, while lower temperature and water pressure favor the latter. For aqueous suspensions at ambient conditions, their coexistence is maximal for particle sizes up to 12 nm. The predictions inform and help resolve different observations in various experiments.

## Abstract (web summary)

Ferrihydrite is the most bioavailable source of ferric iron, but its variable structure and composition are poorly understood because of its small size and lack of ordering. Here ab initio thermodynamics reveals that ferrihydrite can be described as a nanocomposite of at least two previously-proposed single-phase models.

## Introduction

Ferrihydrite is one of the most important, abundant, and enigmatic minerals of the iron-(oxyhydr)oxide family. Typically the first product of Fe(III) hydrolysis or rapid oxidation of aqueous Fe(II), this poorly crystalline nanomineral with a 2–10 nm grain size can be found across a diversity of soil and aquatic environments on Earth^1^. In industry, its high reactive surface area and cost-effectiveness make it a potent sorbent for water purification^2,3^ and catalysis^4^. However, as a poorly ordered material, it has remained difficult for X-ray diffraction (XRD) and scattering techniques to precisely determine its structure, beyond providing an indication of major ferrihydrite types distinguished by the number of peaks present in the XRD pattern. While so-called 2- and 6-line are the most common, ferrihydrite can also exhibit an intermediate number of peaks^1,5–7^. For example, in Si-rich and high-temperature conditions, synthetic and natural 7-line ferrihydrite have been characterized^5,8,9^. Despite these XRD-based distinctions, X-ray pair distribution function analyses suggest that there are no significant structural differences between 2- and 6-line ferrihydrite and that the differences instead reflect the variations in the average size of the coherent scattering domains^7^. This is tacitly consistent with the fact that ferrihydrite exists exclusively as 2–10 nm nanoparticles, mostly spherical in shape, with 2-line ferrihydrite possessing smaller particle sizes (~1–4 nm) and being the most hydrated^7,10–15^.

More than half a century of experimental research on its structure and composition has yet to produce a unanimously accepted model^1,11,16–24^. A variety of structures have been proposed and remain the subject of ongoing scrutiny, including a single-phase^11,16,17,22^, a mixture of defect-free and defective structural units^21,25^, and a hybrid model^26^. A single-phase model entailing 20% tetrahedral iron here termed the Michel model^16^ was later called into question as a non-unique solution to X-ray data by comparing it to a different single-phase structure composed entirely of octahedral iron in the Manceau model^17^. Whether or not tetrahedral iron is present^23,24^ remains an important but uncertain aspect with respect to the unambiguous determination of the structure. This is further complicated by the likelihood that both the structure and properties of ferrihydrite (e.g., magnetism) depend on the composition, particularly water content, and particle size^10,15,27,28^. The only well-accepted features of the various proposed structural models are that ferrihydrite has a hexagonal close-packed anion lattice, is made of a mixture of defective and defect-free structural units, and has variable hydroxyl and water adsorption or incorporation^1,21,22^.

In this regard, it is noteworthy that the structure and composition of ferrihydrite have not been studied systematically as a function of synthesis and storage conditions such as pH and temperature. The slow and spontaneous transformation of ferrihydrite to more stable iron (oxyhydr)oxides such as goethite and hematite are known to depend on both pH and temperature^29,30^. The uncertainties about the structure also make it difficult for computational molecular modeling to rigorously encompass all but the most straightforward single-phase structure models. Consequently, only a limited set of density functional theory (DFT) investigations have been performed to date, focused primarily on the single-phase Michel et al. model^16^, examining its virtual bulk lattice thermodynamics^31,32^ and the role of hydration on structural stability and magnetic properties^28^. The collective observations leave open the possibility that at the nanoscale ferrihydrite is actually an assemblage of coexisting X-ray indistinguishable particles, the distribution of which depends on environmental conditions during formation and that may subsequently evolve over time.

The present study investigates this prospect using DFT calculations adjusted for chemical potentials in aqueous suspension at ambient conditions and taking into account particle surface free energies. The fact that ferrihydrite exists only in nanoparticle form clearly indicates the importance of its surfaces for stabilization. Studies that have examined its size-dependent properties^14,15^ as well as its hypothetical surface structure, reactivity, and surface Gibbs free energies^27,33–36^ suggest that ferrihydrite nanoparticles can be described as having a defect-free, low hydroxyl core consisting of the single-phase model of Michel (Fe_5_O_8_H)^16^ and a more hydrated (i.e., Fe_5_O_8_H + *n*H_2_O) iron-deficient crystallographically-oriented surface region depleted in Fe2 octahedral and Fe3 tetrahedral sites. Using this model, estimation of the Gibbs free energy as a function of the particle size at 298 K and 1 bar indicated that ferrihydrite up to 8 nm in particle size should be stable with respect to hematite^36^. This structural description recently showed good recovery of the size-dependent distribution and density of hydroxyl groups at the surface^37^, though it is unclear the extent to which such a finding should be diagnostic for structure.

By combining similar thermodynamic concepts with DFT calculated energetics in an ab initio thermodynamics (AIT) approach, here we show that ferrihydrite can be described as a nanocomposite of the single-phase models of Michel^16^ and Manceau^17^ that evolves as a function of particle size, temperature, pH, and the partial pressure of water. We show how their relative stabilities evolve in terms of system conditions and increasing particle size that ultimately ends in their conversion to more stable crystalline bulk phases of hematite (α-Fe_2_O_3_), goethite (α-FeOOH), and lepidocrocite (γ-FeOOH). We include in the comparison an orthorhombic phase similar to goethite but with the Fe_5_O_8_H stoichiometry that was predicted to have a low-energy topology competitive with the Michel model though remaining structurally incompatible with the XRD and PDF data of ferrihydrite^28^. We also show how the nanocomposite description connects in specific ways to key aspects of experimental observations. The findings strongly suggest that the ongoing debate about the structure of ferrihydrite can be resolved with a closer examination of sample-specific differences.

## Results and discussion

### Relative phase stability of bulk lattices

**Fig. 1:**
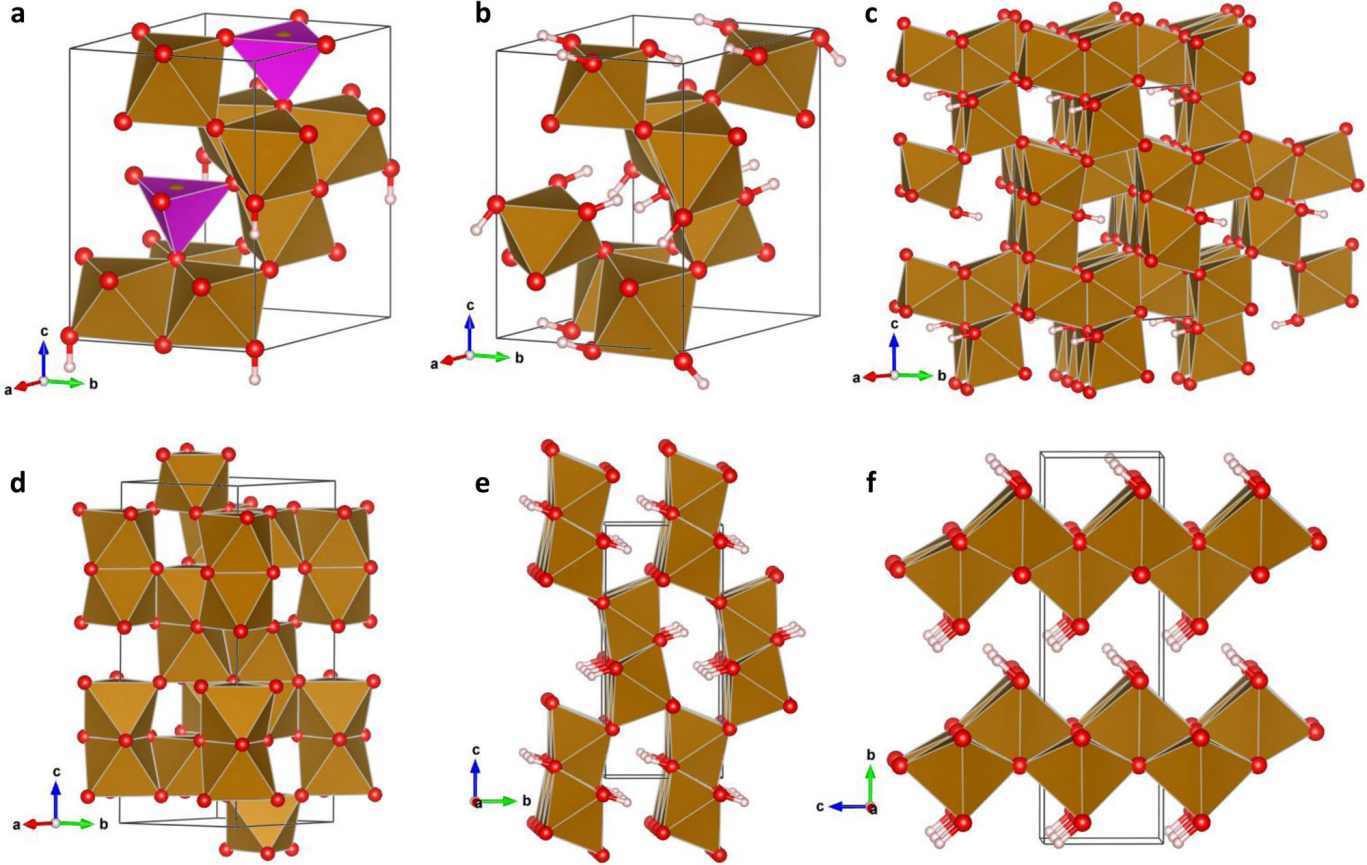
Visual representation of the mineral phases investigated. **a** Michel model for ferrihydrite. **b** Manceau model for ferrihydrite. **c** Orthorhombic phase. **d** Hematite. **e** Goethite. **f** Lepidocrocite.

We first considered virtual bulk structure types of ferrihydrite, taking the single-phase models of Michel^16^ and Manceau^17^, for direct energetic comparison with various Fe(III)-(oxyhydr)oxide phases whose relative stabilities are well known. A visual representation of the mineral phases investigated is shown in Fig. 1.

**Fig. 2:**
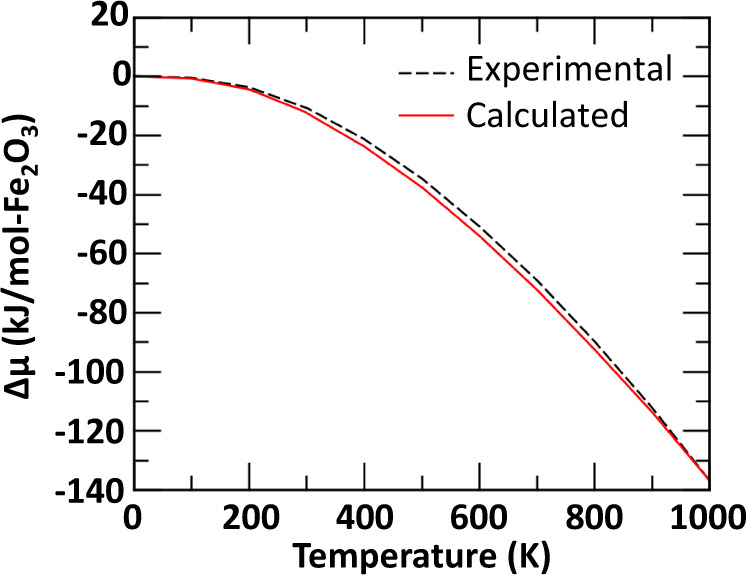
Evolution of the chemical potential with temperature. Comparison between DFT + U calculated and experimental JANAF values of Δ*μ*(*T,P*) (in kJ/mol-Fe_2_O_3_) for hematite (α-Fe_2_O_3_) at *P* = 1 bar.

Following the methodology described in Chaka et al.^38^. we performed DFT-based AIT calculations to predict as a function of temperature and the chemical potential of water the relative phase stabilities of hematite, goethite, lepidocrocite, the two single-phase ferrihydrite models, and the hypothetical orthorhombic phase of Sassi and Rosso^28^. To first establish the accuracy of the approach we examined standard conditions of *P* = 1 bar. The chemical potential, *μ*, of each mineral phase, *i*, was determined by the following equation:

1 $$\mu _i\left( {T,P} \right) = {{{{E}}}}_i^{{{{{{{{\mathrm{Tot,}}}}}}}}\;{{{{{{{\mathrm{0K}}}}}}}}} + {{{{E}}}}_i^{{{{{{{{\mathrm{ZPE}}}}}}}}} + {\Delta} \mu _i\left( {T,P} \right)$$

where *E*_*i*_^Tot, 0K^ is the DFT total energy at 0 K, *E*_*i*_^ZPE^ is the zero-point energy and Δ*μ*_*i*_(*T,P*) the correction to the chemical potential for temperature, *T*, and pressure *P*. In the case of hematite, here taken as the reference mineral phase, the DFT + U calculated values of Δ*μ*(*T,P*), at *P* = 1 bar is in very good agreement, within 3 kJ/mol-Fe_2_O_3_, with experiment^39^ as shown in Fig. 2.

Depending on the stoichiometry of each mineral phase, the Gibbs free energy at standard (*T*,*P*) conditions, Δ*G*°(*T*,*P*), expressed in kJ/mol-FeO_3/2_, was calculated for reactions (2) and (3) according to relationships (4) and (5) respectively, in which each *μ*_*i*_ was calculated as defined by Eq. (1):

2 $$2\ {{{{\mathrm{FeOOH}}}}} \to \alpha\mbox{-} {{{{\mathrm{Fe}}}}}_2{{{{\mathrm{O}}}}}_3 + {{{{\mathrm{H}}}}}_2{{{{\mathrm{O}}}}}$$

3 $$2\ {{{{\mathrm{Fe}}}}}_5{{{{\mathrm{O}}}}}_8{{{{\mathrm{H}}}}} \to 5\left[ {\alpha\mbox{-} {{{{\mathrm{Fe}}}}}_2{{{{\mathrm{O}}}}}_3} \right] + {{{{\mathrm{H}}}}}_2{{{{\mathrm{O}}}}}$$

4 $${{\Delta} G^\circ \left( {T,P} \right) = \mu _{{{{{\mathrm{FeOOH}}}}}}\left( {T,P} \right) - \frac{1}{2}\big[ {\mu _{\alpha\mbox{-} {{{\mathrm{Fe}}}}_2{{{\mathrm{O}}}}_3}\left( {T,P} \right) + \mu _{{{{\mathrm{H}}}}_{2}{{{\mathrm{O}}}}}\left( {T,P} \right)}\big]}$$

5 $${\Delta} G^ \circ \left( {T,P} \right) = \frac{1}{5}\mu _{{{{{{{\rm{Fe}}}}}}_5{{{{{\rm{O}}}}}}_8{{{{{\rm{H}}}}}}}}\left( {T,P} \right) - \frac{1}{2}\mu _{\alpha\mbox{-} {{{{{{\rm{Fe}}}}}}_2{{{{{\rm{O}}}}}}}_3}\left( {T,P} \right) - \frac{1}{{10}}\mu _{{{{{{{\rm{H}}}}}}_2{{{{{\rm{O}}}}}}}}\left( {T,P} \right)$$

At standard conditions (*T* = 298.15 K and *P* = *p*H_2_O = 1 bar) the value of *ΔG°* obtained for Eqs. (4) and (5) for hematite, goethite, lepidocrocite, the two ferrihydrite models, and the orthorhombic phase are shown in Table 1. As in previous^31^ theoretical calculations of *ΔG°* between bulk hematite and goethite, our value of the Gibbs free energy at these (*T*,*P*) conditions predicts that goethite should be slightly more energetically favorable than hematite. While inconsistent with the assertion from experiments^15,40^, the absolute error of the calculated *ΔG°* remains small, about 3 kJ/mol-FeO_3/2_. Furthermore, if we consider instead the partial pressure of water in equilibrium with liquid water at ambient conditions (*T* = 298.15 K and *p*H_2_O = 32 mbar), we now find that goethite is slightly less favorable than hematite by about 0.9 kJ/mol-FeO_3/2_ in good agreement with experiments^15,40^. The calculated Gibbs free energy of lepidocrocite, relative to hematite, is close to the experimental value for both partial pressures of water.

**Table 1:** Gibbs free energies of bulk mineral phases.

Structure	${\Delta} G_{{{{{{{{\mathrm{GGA + U}}}}}}}}}^ \circ p{{{{{{{\mathrm{H}}}}}}}}_2{{{{{{{\mathrm{O}}}}}}}}$ = 1 bar	${\Delta} G_{{{{{{{{\mathrm{GGA + U}}}}}}}}}^ \circ p{{{{{{{\mathrm{H}}}}}}}}_2{{{{{{{\mathrm{O}}}}}}}}$ = 32 mbar	${\Delta}{G^ {\circ}_{{{\mathrm{GGA+U}}}}}$^30^	${\Delta} G_{{{{{{{{\mathrm{Exp}}}}}}}}}^ \circ$^15,40^
Hematite (α-Fe_2_O_3_)	0.00	0.00	0.00	0.00 ± 0.60
Goethite (α-FeOOH)	−3.34	0.93	−1.50	0.16 ± 0.80
Lepidocrocite (γ-FeOOH)	7.22	11.49	10.20	8.06 ± 2.40
Manceau model (FeOOH)	0.09	4.36	n/a	16.90–22.70 ± 1.20
Michel model (Fe_5_O_8_H)	4.63	5.48	6.90	16.90–22.70 ± 1.20
Orthorhombic (Fe_5_O_8_H)	7.52	8.37	n/a	n/a

Calculated Gibbs free energies (in kJ/mol-FeO_3/2_) with respect to ½ [α-Fe_2_O_3_] at *T* = 298.15 K and two water partial pressures (*p*H_2_O).

For the Michel ferrihydrite model, our calculated Gibbs free energies are in good agreement with previous DFT calculations^31^. However, all such calculations are not readily compared to experiments because they do not take into account the energy cost associated with the surface energy of ferrihydrite nanoparticles^31^. We also note that the experimentally determined *ΔG°* values were calculated using the assumption that ferrihydrite has the stoichiometry Fe(OH)_3_, which is not the case for the Michel (Fe_5_O_8_H) and Manceau (FeOOH) models being considered here. The use of the highly hydrous formula Fe(OH)_3_ in the literature stems from the difficulty to precisely distinguish structural versus physisorbed water in experimental characterization studies. As shown in Table 1, the comparison of *ΔG°* values obtained at *T* = 298.15 K and *p*H_2_O = 1 bar for the Manceau and Michel models suggest that the Manceau phase should form preferably because it is 4.54 kJ/mol-FeO_3/2_ more energetically favorable than the Michel model, at least in the hypothetical bulk crystal structure form. For *p*H_2_O = 32 mbar which is the vapor pressure of liquid water, the Manceau model is still more favorable than the Michel model but the energetic difference is smaller, only 1.12 kJ/mol-FeO_3/2_. Table 1 also shows that the orthorhombic phase is only 2.89 kJ/mol-FeO_3/2_ less favorable than the Michel model at both *p*H_2_O.

**Fig. 3:**
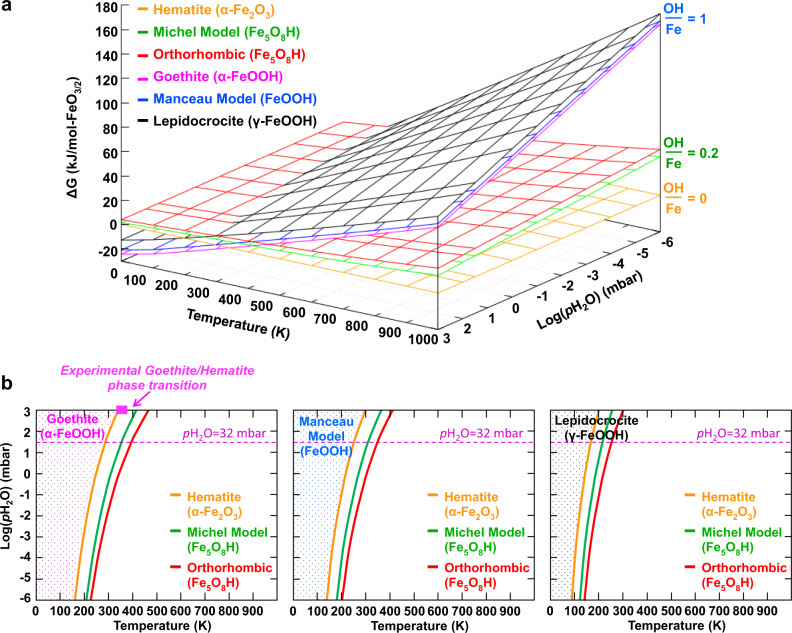
Relative phase stability of mineral phases. Calculated relative bulk phase stabilities for hematite, goethite, lepidocrocite, the two ferrihydrite models, and the orthorhombic structure. **a** 3D plot of the Gibbs free energy as a function of the temperature and water partial pressure. **b** Water pressure-temperature phase diagrams showing the domain of stability for each phase and the conditions at various phase boundaries. The purple dashed line indicates the water pressure for which *p*H_2_O = 32 mbar. The color dotted areas highlight the (*T*,*P*) conditions at which each FeOOH phase is the most thermodynamically favorable.

To examine the role of water chemical potential on relative phase stabilities, we calculated *ΔG* over a broad range of hypothetical temperature (0–1000 K) and water partial pressure (10^−6^–10^3^ mbar), using hematite as the reference phase (*ΔG* = 0 kJ/mol-FeO_3/2_) because it is independent of the water chemical potential (i.e., OH/Fe = 0). As shown in Fig. 3a, compared to hematite the next most “dry” phases are the Michel ferrihydrite model and the orthorhombic phase, both of which possess the same Fe_5_O_8_H stoichiometry. The Michel model is always less favorable than hematite, and the orthorhombic phase is always less favorable than the Michel model, with both following a very similar free energy planarity to hematite because of their low molar water contents of OH/Fe = 0.2. At higher molar water contents of OH/Fe = 1.0 are the phases goethite, lepidocrocite, and the Manceau ferrihydrite model, each having the FeOOH stoichiometry and each following similar planarity but with a much higher dependence on the chemical potential of water collectively. Regardless of the (*T*,*P*) conditions, our calculations predict that the Manceau model is less favorable than goethite and always more favorable than lepidocrocite.

Phase crossover regions among these three major groups of phases (OH/Fe = 0, 0.2, and 1.0) provide initial clues into the prospect of phase coexistence at select (*T*,*P*) conditions. At the reference state of *p*H_2_O = 1 bar, the experimental temperature for the goethite-hematite phase transition is in the range of 340–370 K^1,15^, which is satisfactorily reproduced by our calculated temperature of 340 K (Fig. 3b). At *T* = 300 K and *p*H_2_O = 1 bar, the calculations predict that the Manceau ferrihydrite model is more stable than the Michel model, though they become thermodynamically isoenergetic at the mildly elevated *T* = 360 K. This suggests that the presence of tetrahedral iron in the core of ferrihydrite nanoparticles, as present in the single-phase Michel model, could be favored at mildly elevated temperature. In comparing the domains of stability for the Michel and Manceau ferrihydrite models, the results in Fig. 3b reinforce a picture that the Michel model could comprise a relatively dry polymorph of ferrihydrite, while the Manceau model could comprise a wetter polymorph, with temperature as an important determining variable within a few degrees.

### Nanoparticle thermodynamics and coexistence

**Fig. 4:**
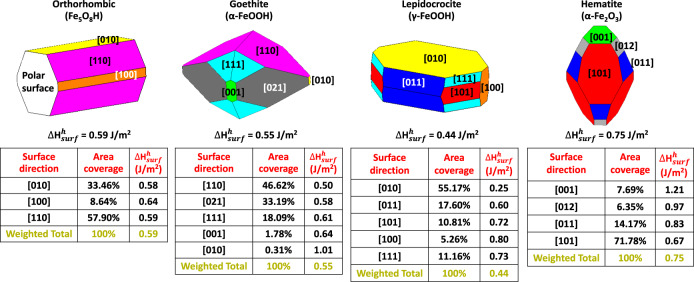
Wulff representations and hydroxylated surface enthalpies of iron-(oxyhydr)oxide minerals. Morphology averaged surface enthalpies (${\Delta} {{{H}}}_{surf}^h$) of the mineral investigated. The surface enthalpies of the two ferrihydrite models have been assumed to vary between 0.10 and 0.40 J/m^2^.

Because of the clear importance of including surface free energy contributions towards understanding relative phase stabilities at the nanoscale^41–43^, we extended the AIT calculations to consider the effects of finite particle size. The estimation of the surface energy contribution at finite particle size requires the explicit calculation of the surface enthalpy and entropy. The determination of hydroxylated surface enthalpy (${\Delta} {{{{H}}}}_{surf}^h$) used empirical potentials and the Metadise code^44^ which automatically detects polar surfaces finds the lowest energy plane were to cleave bulk structures and constructs a semi-infinite interface to model a surface. For each mineral phase, the calculations of surface enthalpies were performed in several directions by following the methodology outlined by de Leeuw et al.^45^. Surface directions with low Miller indices were preferentially selected and the calculated surface enthalpy for each cleaved and hydroxylated surface is summarized in Table S1 in the Supplementary Information. These enthalpies were calculated using the following equations:

6 $${\Delta} {{{{H}}}}_{surf}^p = \frac{{E_{surf - pure} - E_b}}{A}$$

7 $${\Delta} {{{{H}}}}_{surf}^h = \frac{{E_{surf - hydro} + nE_{diss - water} - E_{surf - pure}}}{A} + {\Delta} {{{{H}}}}_{surf}^p$$

where ${\Delta} {{{{H}}}}_{surf}^p$ and ${\Delta} {{{{H}}}}_{surf}^h$ are respectively the surface enthalpy of a cleaved and hydroxylated surface, *A* is the surface area, $E_{surf - pure}$ and $E_{surf - hydro}$ are respectively the calculated energy of the cleaved and hydroxylated surface, *E*_*b*_ is the energy of the bulk mineral structure, *n* and $E_{diss - water}$ are respectively the number of water molecules used for surface hydroxylation and the water dissociation energy. As shown in Fig. 4, using empirical potentials and explicitly described atomistic surface terminations we calculated a morphology weighted averaged hydroxylated surface energy for hematite, goethite, and lepidocrocite based on their Wulff construction, and found that for these three mineral phases ${\Delta} {{{{H}}}}_{surf}^h$ is 0.75 J/m^2^, 0.55 J/m^2^, and 0.44 J/m^2^, respectively. These ${\Delta} {{{{H}}}}_{surf}^h$ values are in very good agreement with the experimentally determined surface energy of hematite (0.75 J/m^2^), goethite (0.60 J/m^2^), and lepidocrocite (0.40 J/m^2^)^15^. The estimated ${\Delta} {{{{H}}}}_{surf}^h$ for the orthorhombic phase, 0.59 J/m^2^, is close to the one of goethite, which is not surprising given the fact that these two phases share some similar structural features^28^. In contrast to these well-ordered phases, performing the same kind of explicitly described surface calculations to determine the weighted averaged hydroxylated surface energies of the Manceau and Michel ferrihydrite models would be of questionable value due to expectedly important irregularities in particle shape, surface stoichiometry and reconstruction, and defects. Indeed, the use of the defect-free model structures to calculate hydroxylated surface enthalpies, as shown by Table S1 in Supplementary Information, suggests that many surface orientations for the Michel model are electrostatically unstable and have a dipole, which indicates that surface reconstructions are very likely to occur. Table S1 also shows that regardless of the surface orientation, both ferrihydrite models have their calculated hydroxylated surface enthalpies larger than 0.40 J/m^2^, which is the lowest surface enthalpy experimentally measured by calorimetry for lepidocrocite^15^. This indicates that a Wulff construction based on these surface enthalpies will lead to an average surface enthalpy larger than 0.40 J/m^2^. While the method used to calculate surface enthalpies for the other (more stable) mineral phases provide values in very good agreement with experiments, the results obtained for the ferrihydrite models using cleaved, defect-free hydroxylated surfaces do not seem realistic as experiments tend to indicate that the surfaces of ferrihydrite are highly defective and that ferrihydrite nanoparticles have a low surface free energy given its metastability and thermodynamics compared to other more stable iron-oxyhydroxides. Based on this analysis, we therefore instead rely on a range of reasonable surface energies for the two ferrihydrite models, as discussed by Pinney et al.^31^. We assume that the surface energy of ferrihydrite can vary in a range of values, from the lowest 0.10 J/m^2^, as determined by Hiemstra^36^, to the highest 0.40 J/m^2^, the experimentally determined surface energy of lepidocrocite^15^.

**Fig. 5:**
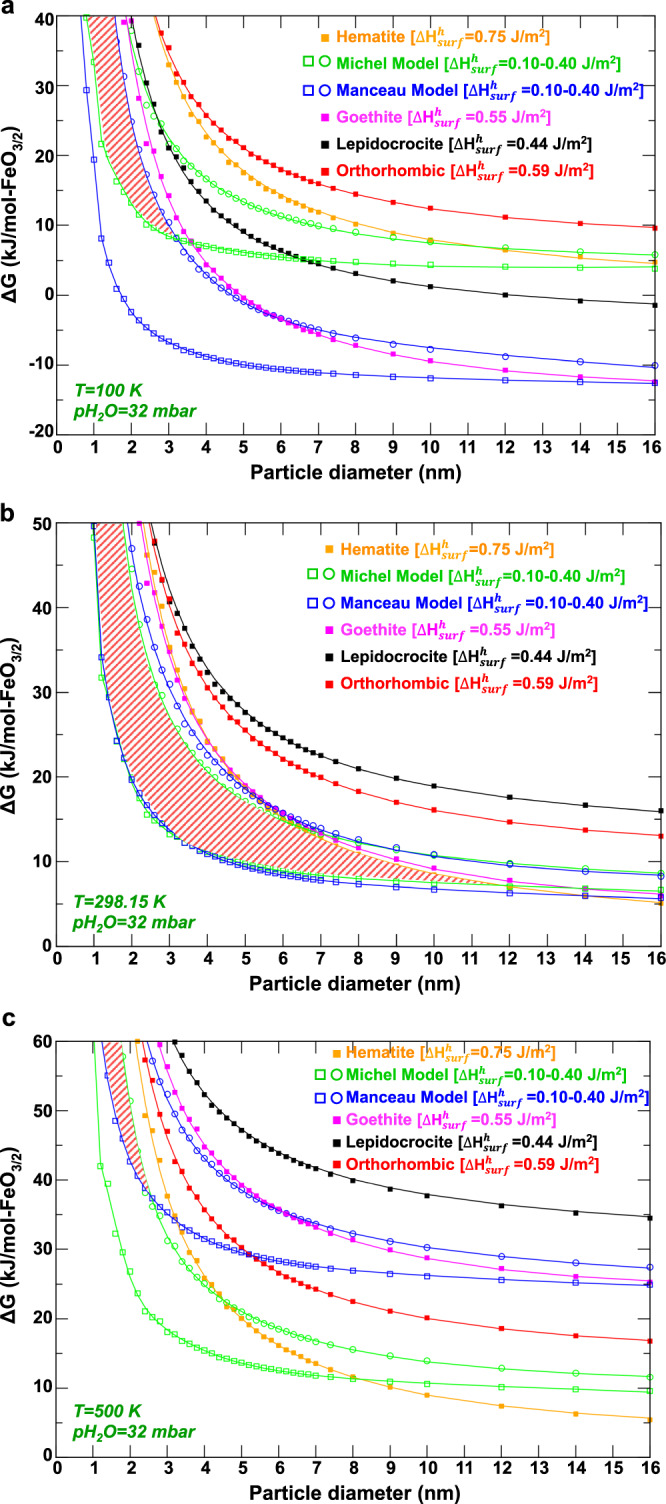
Coexistence of structurally different Fh nanoparticles. Particle-size dependency of the relative phase stability of the Manceau and Michel ferrihydrite models as well as for goethite, lepidocrocite, hematite, and the orthorhombic phase for *p*H_2_O = 32 mbar and (**a**) *T* = 100 K, (**b**) *T* = 298.15 K, and (**c**) *T* = 500 K. For the two ferrihydrite models, the curves with open squares were obtained using ${\Delta} {{{{H}}}}_{surf}^h$ = 0.10 J/m^2^, while the curves with open circles used ${\Delta} {{{{H}}}}_{surf}^h$ = 0.40 J/m^2^. Square filled curves used ${\Delta} {{{{H}}}}_{surf}^h$ calculated for hydroxylated periodic surface crystals. The red hatched area represents the size range of particles for which the two ferrihydrite models can potentially coexist.

Relative phase stabilities for the six mineral phases of interest as a function of particle size were computed for *p*H_2_O = 32 mbar, and three different temperatures of *T* = 100 K, 298.15 K, and 500 K, variation of which enables insight into the completeness of the overlapping stability fields for the two types of studied ferrihydrite nanoparticles (Fig. 5). A comparison between *p*H_2_O = 32 mbar and *p*H_2_O = 1 bar results is given in Fig. S1. The curves with open squares are those obtained for ferrihydrite nanoparticles with a minimal surface energy of 0.10 J/m^2^, while the curves with open circles are calculated using surface energy of 0.40 J/m^2^. In the following, ferrihydrite nanoparticles with a structure based on the Michel model will be referred to as the Michel ferrihydrite nanoparticles and, likewise for those of the Manceau. Relative phases stabilities can now be discussed in terms of particle size dependency, over the evaluated size range of ~0 to 16 nm in diameter.

As shown in Fig. 5a, at a temperature of 100 K the lowest energy phase is predicted to be Manceau ferrihydrite particles over the entire size range examined when assigned their lowest surface free energy (0.10 J/m^2^). Manceau particles at their highest surface free energy (0.40 J/m^2^) follow closely with goethite particles as the two next lowest free energy phases over most of the size range. This suggests that at this low-temperature Manceau ferrihydrite nanoparticles would become unstable with respect to goethite when their diameter increases above the range from 6  nm to 16 nm. At 100 K, no ferrihydrite particles should be expected with a diameter above 16 nm. Importantly, a comparison between Michel and Manceau ferrihydrite nanoparticles suggests that the former could only be stable below 3.2 nm when assigned its lowest surface free energy, compared to the highest surface free energy Manceau phase. In this case, hypothetically, if chemical conditions were such that the exchange of one Fe^3+^ for three H^+^ was possible at no energetic cost (i.e., to enable FeO_5_H_8_ + 2H_2_O = FeOOH) then Michel ferrihydrite nanoparticles with diameters larger than 3.2 nm would transform into Manceau ferrihydrite nanoparticles instead of transforming into 3.6 nm goethite; the red hatched area in Fig. 5a highlights that at 100 K coexistence of the Manceau and Michel ferrihydrite nanoparticles is possible up to about 3.2 nm in size.

At room temperature, the coexistence field between Michel and Manceau ferrihydrite nanoparticles is predicted to be much larger and unconstrained by their surface energies, as shown in Fig. 5b. At 298.15 K, the Manceau and Michel ferrihydrite nanoparticles are effectively isoenergetic up to particle diameters up to ~5 nm, after which these particles become increasingly unstable with respect to hematite. A destabilization sequence can be defined in terms of specific ferrihydrite phase and surface free energy. The highest surface free energy Manceau and Michel ferrihydrite nanoparticles would start to transform into hematite first, followed by the lowest surface free energy Michel particles at >11.8 nm, and ultimately the lowest surface free energy Manceau particles at diameters >14 nm. Here again, no ferrihydrite nanoparticles should be observable with diameters larger than 16 nm, consistent with expectations. And for *p*H_2_O = 1 bar, the Michel ferrihydrite nanoparticles should be stable with respect to goethite up to 8 nm (Fig. S1e), in good agreement with the previous calculations of Hiemstra^36^.

From the large size of the coexistence field in Fig. 5b, a major finding of our study is the likelihood of structural diversity of ferrihydrite nanoparticles at room temperature in aqueous suspension, with at least two single-phase ferrihydrite structure types predicted to be energetically favorable simultaneously over a fairly large particle size distribution. Considering the ongoing debate across experimental studies about the structure of ferrihydrite, which in large part is based on the challenges in obtaining a unique structural signature of ferrihydrite based on X-ray diffraction and scattering analyses, this finding is particularly relevant.

Also, at room temperature, we remark that the orthorhombic phase^28^ is predicted to behave very similarly to lepidocrocite across the whole range of particle sizes investigated. For *p*H_2_O = 32 mbar, the orthorhombic phase is found to be energetically more favorable than lepidocrocite at all particle sizes investigated. Although there is no indication from experiments that such an orthorhombic phase exists, this finding would suggest that if it did exist it could be maintained at very low concentrations as the transient intermediate during the transformation of Michel ferrihydrite particles to goethite. The prospect of this phase potentially mediating a solid-state transformation pathway will be explored in future modeling work.

At the highest evaluated temperature of 500 K, the formation of hematite is preferred over goethite and the Michel ferrihydrite nanoparticles are generally more energetically favorable than the Manceau ferrihydrite nanoparticles (Fig. 5c). As indicated by what now again is a relatively small coexistence field similar to that at 100 K, the two ferrihydrite structure types coexist over a small range of diameters, up 2.6 nm, beyond which the Michel ferrihydrite nanoparticles should be the only ferrihydrite structure present. Similar to the case at 100 K but in the opposite sense, if iron and proton exchanges are possible then Manceau ferrihydrite nanoparticles with a diameter larger than 2.6 nm should be able to transform into Michel ferrihydrite nanoparticles prior to transformation into hematite. At 500 K, Michel ferrihydrite nanoparticles should start to transform into hematite if their diameter is larger than 4.5 nm and no ferrihydrite should be observable for nanoparticles with a diameter larger than 8.2 nm.

### Connection to experimental data

It is possible to connect our nanocomposite prediction to the hydrothermal aging experiment of Michel et al.^14^, who monitored the evolution of average particle size and composition as a function of aging time during 14 h at 175 °C (473 K) (see Tables S2 and S3 in Supplementary Information of Michel et al.^14^). Starting from an initial (*t* *=* 0 h) composition and average particle size of Fe_8.2_O_15.9_H_7.4_·3H_2_O and 2.28 nm, respectively, they observed a progressive decrease in water content and increase in average particle size over aging time (*t* *=* 3 h: Fe_8.6_O_16.1_H_6.2_·2.5H_2_O and 3.55  nm; *t* = 8 h: Fe_10_O_16_H_2_·1.2H_2_O and 6.8 nm; *t* *=* 11 h: Fe_10_O_16_H_2_·0.5H_2_O and 9.06 nm). Ferrihydrite was the only phase observed from *t* = 0–7 h whereas from *t* = 7–12 h ferrihydrite and an increasing amount of hematite was observed until only hematite remained at *t* = 14 h. This suggests a sequence that progresses from an initially hydrous small particle size ferrihydrite to one of larger diameter containing less water that ultimately is consumed by conversion to hematite nanoparticles.

The same sequence emerges from our calculations at the nearest comparable temperature of 500 K (Fig. 5c) if one assumes that the initial ferrihydrite is comprised of a coexisting assemblage of the relatively hydrous Manceau (Fe_10_O_16_H_2_) particles and relatively dry Michel (Fe_10_O_16_H_2_) particles coexisting up to diameters of 2.6 nm (i.e., up to *t* *≈* *2* h of experimental time). The initial predominance of the Manceau particles would be consistent with the observed more hydrous initial composition, particularly Manceau particles with a slight deficiency of iron charge-balanced by an excess of protons, such as by protonation of lattice O^2−^ sites accessible within 2 × 1 goethite-like channel. (Such a condition would likewise destabilize the Michel structure, as shown by Pinney et al.^32^). In addition, the experimental lattice parameters at *t* *=* 0 h, *a* = 5.96 Å, and *c* = 9.02 Å (see Table S1 in Supplementary Information of Michel et al.^14^), are much closer to the DFT calculated lattice parameters we obtained for the Manceau model, *a* = 5.97 Å and *c* = 9.00 Å than to the calculated lattice parameters for the Michel model, *a* = 5.87 Å and *c* = 9.37 Å^28^. As this assemblage ages with temperature and average particle sizes increase, Manceau particles would diminish first and shift the distribution towards Michel particles (Fig. 5c). The experimental observation that hematite is increasingly detected when the average ferrihydrite nanoparticles size range from 8.79 nm (*t* = 9 h) to 10.96 nm (*t* = 12 h) is in good agreement with our predicted maximal particle size diameter after which the Michel ferrihydrite nanoparticles become unstable with respect to hematite (>8.2 nm in Fig. 5c). In addition, the experimental composition analysis of ferrihydrite from *t* *=* 8 h to *t* *=* 12 h (Fe_10_O_16_H_2_·*x*H_2_O) suggests that only the Michel ferrihydrite nanoparticles are present, possessing a particle size ranging from 6.80 nm to 10.96 nm, in good agreement with our theoretical results.

### Effect of pH on iron-oxyhydroxide solubility

**Fig. 6:**
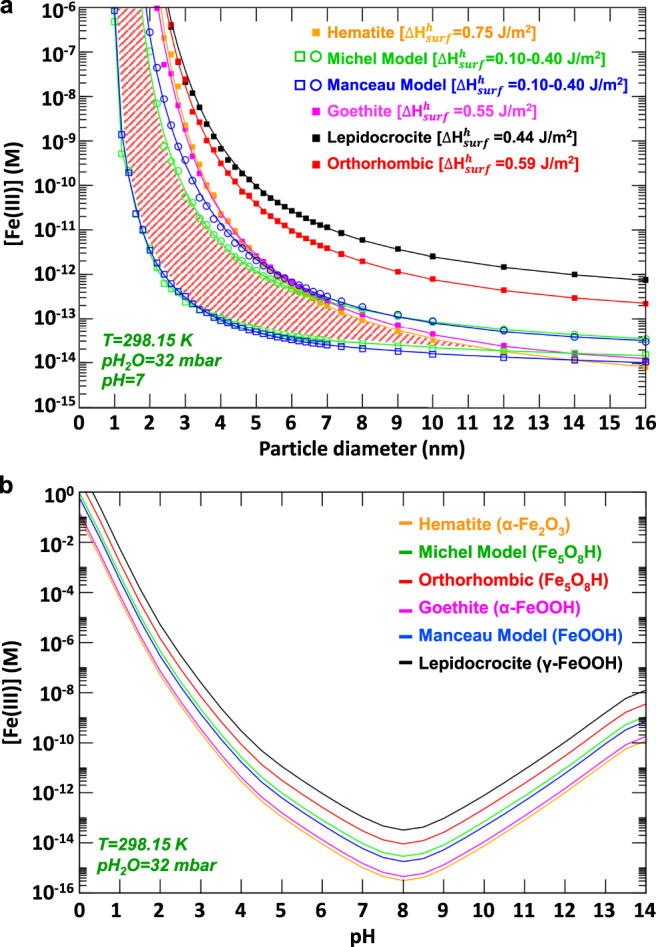
Solubility of iron-oxyhydroxide phases. Equilibrium solubility of [Fe(III)] for the various minerals in pure water. **a** Particle-size dependence of [Fe(III)] solubility at pH = 7. The red hatched area represents the size range of particles for which the two ferrihydrite models can potentially coexist. **b** pH-dependence of [Fe(III)] solubility of iron (oxyhydr)oxide phases considered as virtual bulk lattices.

In addition to temperature and water chemical potential, pH is also a key parameter affecting the chemical behavior of ferrihydrites such as its conversion rate to goethite and hematite^29,30^. To evaluate the effect of pH on relative nanoparticle phase stabilities, we used the AIT calculated Gibbs free energies in combination with the Ostwald equation^46^ and experimental hydrolysis reaction rates^47,48^ to estimate equilibrium [Fe(III)] solubility for each mineral phase investigated. At *T* = 298.15 K, *p*H_2_O = 32 mbar, and pH = 7, Fig. 6a shows that the relative stability of the various phases in terms of solubilities is similar to those observed in Fig. 5b across a wide pH range. In contrast to the particle-size effect, which depends primarily on different (T, *p*H_2_O) conditions, the main effect of pH is to shift the equilibrium [Fe(III)] solubility toward either higher or lower values for all phases (Figs. 6b and S2). Figure 6b shows an asymmetric pH-dependent solubility which is lowest at pH = 8 for each mineral phase. Importantly, in terms of relative [Fe(III)] solubilities, the findings predict an order of appearance (e.g., from saturated aqueous solution) analogous to an Ostwald–Lussac law of phases of lepidocrocite → orthorhombic phase → Michel Fh → Manceau Fh → goethite → hematite. The close [Fe(III)] solubilities predicted for the two ferrihydrite models of Michel and Manceau resembles the same close relationship between hematite and goethite.

## Conclusions

DFT-based ab initio thermodynamics calculations comparing the stabilities of two prominent single-phase structure models of ferrihydrite in aqueous environments indicate overlapping phase fields and their likely coexistence at the nanoscale. The finding suggests that phases are known as 2-line and 6-line ferrihydrites may actually be comprised of an assemblage of distinct particle types not readily distinguishable by X-ray diffraction or scattering techniques.

In terms of virtual bulk lattices, analysis of the relative phase stability indicates that the Manceau model is stable for temperatures below 295 K with respect to hematite and is the preferred ferrihydrite phase at standard temperature and pressure (i.e., *T* = 298.15 K and *p*H_2_O = 1 bar) compared to the Michel model. The Manceau model corresponds to a wet ferrihydrite phase. In contrast, the Michel model is stable at temperatures above 360 K and can be considered the most suitable model for a dry phase of ferrihydrite. A hypothetical orthorhombic phase was found to be only 0.3 kJ/mol-Fe less favorable than lepidocrocite at standard temperature and pressure, representing a possible transition phase to goethite that is competitive with lepidocrocite. The predicted best temperature range to stabilize this orthorhombic structure is between 303 K and 340 K, which is just after the lepidocrocite/orthorhombic phase transition and just before the goethite/hematite phase transition.

Calculation of particle size-dependent ab initio thermodynamics indicates that the Manceau and Michel single-phase ferrihydrite nanoparticles can be expected to generally coexist for small particle diameters at least up to approximately 3 nm over a large working range of temperature and partial pressure of water. The main effect of temperature and water pressure is to shift the distribution of ferrihydrite particle types via differences in their stable particle sizes, and also to determine the most stable crystalline end-product phase into which they transform. Higher temperature and/or higher water pressure favor the formation of Michel ferrihydrite nanoparticles and hematite, while lower temperature and/or lower water pressure favor the formation of Manceau ferrihydrite nanoparticles and goethite. We found that at the routine experimental conditions of 298.15 K and *p*H_2_O = 1 bar both wet and dry single-phase ferrihydrite particle types can be expected to coexist up to about 8 nm in particle diameter.

## Methods

### DFT simulations

Ab initio total-energy calculations and geometry optimizations were performed using the VASP package^49,50^. All of the calculations used the generalized gradient approximation (GGA), as parametrized by Perdew, Burke, and Ernzerhof (PBE)^51^, to describe the exchange-correlation functional. The cutoff energy of the projector augmented wave^52^ pseudo-potentials were all fixed to 800 eV. In each case, the total energy was converged to 10^−8^ eV/cell and the force components were relaxed to 10^−5^ eV/Å. A Monkhorst–Pack scheme *k*-points mesh of 6 × 6 × 4 was used for the sampling of the Brillouin zone. In order to correctly describe the magnetic ordering of the ground state of the six iron-oxide phases of interest, each calculation used spin-polarization. In addition, the GGA + U method, as described by Dudarev^53^, was used for the Fe atoms to correct the poor description of the Coulomb repulsion of the 3d electrons^54^ in standard GGA. The Hubbard parameter *U*, describing the Coulomb interaction, was fixed to 5 eV, while the screened exchange energy, *J*, was fixed to 1 eV (*U*_*eff*_ = *U* *–* *J* = 4 eV). The *U*_*eff*_ value was selected to reproduce the correct relative phase stability between hematite and goethite at ambient conditions (i.e., *T* = 298.15 K and *p*H_2_O = 32 mbar). It was also found that this value provides a good description of the chemical potential evolution of hematite as function of temperature, as shown in Fig. 2. In order to account for the atomization errors induced by GGA for H_2_O, H_2_, O_2_, and Fe the total energies of these species have been corrected by using their experimental, zero-point energy corrected, atomization energies^51,55^. Therefore, the total energy of H_2_O, H_2_, O_2_ molecules, and Fe bulk were respectively corrected by 0.029 eV, −0.192 eV, 0.805 eV, and −0.495 eV.

### Phonon dispersion relationship

The phonon dispersion relationship was computed for each mineral phase by using a direct approach to the lattice dynamics, as implemented in the phonopy code^56^. The vibrational frequencies, obtained at the Γ-point, were subsequently used to calculate the zero-point energy of each phase as well as the temperature dependent vibrational entropy contribution. These two quantities were used to calculate AIT quantities of periodic bulk and nanoparticles.

### Hydroxylated surface enthalpy and entropy

Including the effect of particle size in the AIT calculations required evaluation of the surface entropy and enthalpy contributions. The surface enthalpy contributions for goethite, hematite, lepidocrocite, and the orthorhombic phase, were treated explicitly, using classical force field methods as implemented in the Metadise code^44^. For each of these mineral phases, at least seven surface directions were investigated and the calculation of hydroxylated surface enthalpy was determined as described by de Leeuw et al.^45^, which uses a calculated water dissociation energy. The force field for each species used in these classical simulations was the same as those listed in that study^45^. The lowest hydroxylated surface enthalpy calculated for each surface direction was used to build the Wulff construction for each mineral phase, representing the equilibrium crystal morphology for hydroxylated surfaces, by using the Wulffman program^57^. From the Wulff construction, we determined a weighted averaged hydroxylated surface enthalpy, ${\Delta} {{{{H}}}}_{surf}^h$.

### Nanoparticle thermodynamic model

For the two ferrihydrite models, goethite, hematite, lepidocrocite, and the orthorhombic phases, the determination of the surface entropy contributions was performed as described in the thermodynamical framework of Hiesmtra^36^. For goethite, hematite, lepidocrocite, and the Michel ferrihydrite model, contributions similar to those of Hiemstra^36^ were obtained for the surface entropy. The details of each contribution to the surface entropy can be found in Tables S2 and S3 in the Supplementary Information. The calculation of pH-dependent [Fe(III)] solubilities for each mineral phase followed the methodology outlined by Hiemstra^36^, Liu^47^, and Millero^48^. Additional details on the calculation of the solubilities are provided in Table S4 in Supplementary Information.

## Supplementary information

Supplementary Information

## Data Availability

Any relevant data are available from the authors upon reasonable request.
