# Supplementary material for: Ab initio thermodynamics reveals the nanocomposite structure of ferrihydrite
Source: Commun Chem. 2021 Sep 20;4:134. doi: 10.1038/s42004-021-00562-7 (PMC9814694; doi:10.1038/s42004-021-00562-7)
Supplement: Supplementary file 1 — Supplementary Information [file 42004_2021_562_MOESM1_ESM.pdf]

Supplementary Information for

Ab Initio Thermodynamics Reveals the Nanocomposite Structure of Ferrihydrite

Michel Sassi[1,*], Anne M. Chaka[1], and Kevin M. Rosso[1]

[1]Physical and Computational Sciences Directorate, Pacific Northwest National Laboratory, Richland, Washington 99352, USA

*E-mail: michel.sassi@pnnl.gov

CONTENT

Details of surface enthalpies calculated with Metadise p2
Surface entropy and temperature-dependent surface energy p3
Effect of different (T,pH_2O) conditions on relative phase stability p4
AIT calculated Gibbs free energy and equilibrium reactions p5
Equilibrium solubility and hydrolysis reactions p5
Effect of pH on the solubility of [Fe(III)] of iron-(oxyhydr)oxide minerals p6
Supplementary References p7

- **Details of surface enthalpies calculated with Metadise:**

Surface direction	Goethite		Hematite		Lepidocrocite		Orthorhombic phase		Michel Model		Manceau Model	
	ΔH^{p}_{surf} (J/m^2)	ΔH^{h}_{surf} (J/m^2)	ΔH^{p}_{surf} (J/m^2)	ΔH^{h}_{surf} (J/m^2)	ΔH^{p}_{surf} (J/m^2)	ΔH^{h}_{surf} (J/m^2)	ΔH^{p}_{surf} (J/m^2)	ΔH^{h}_{surf} (J/m^2)	ΔH^{p}_{surf} (J/m^2)	ΔH^{h}_{surf} (J/m^2)	ΔH^{p}_{surf} (J/m^2)	ΔH^{h}_{surf} (J/m^2)
[001]	1.68	0.64	1.78	1.21	0.93	0.69	dipole	-	dipole	-	1.70	0.71
[100]	1.48	0.78	1.99	1.01	1.46	0.80	1.81	0.64	1.42	0.56	1.12	0.60
[010]	0.68	1.01	1.99	1.01	0.25	0.25	1.86	0.58	1.80	0.54	0.58	1.16
[011]	1.86	0.64	2.41	0.83	0.81	0.60	dipole	-	dipole	-	1.39	0.74
[110]	1.18	0.50	2.03	0.81	1.49	0.84	1.40	0.59	1.73	0.81	0.96	0.72
[101]	1.32	0.84	2.34	0.67	1.68	0.72	dipole	-	dipole	-	1.64	0.44
[111]	1.33	0.61	2.41	0.84	1.66	0.73	dipole	-	dipole	-	1.38	0.75
[021]	1.22	0.58									1.13	0.59
[012]			2.36	0.97								
[102]			1.88	0.97								

Table S1: Calculated surface enthalpies of cleaved (ΔH^{p}_{surf}) and hydroxylated (ΔH^{h}_{surf}) surfaces for goethite, hematite, lepidocrocite, the orthorhombic phase, and the two ferrihydrite models.

- **Surface entropy and temperature-dependent surface energy:**

Mineral phases	ρ_{H_2O} (μmol/m^2)	$S_{H_2O}^{Chem}$ (J.mol^{-1}.K^{-1})	$S_{H_2O}^{Phys}$ (J.m^{-2}.K^{-1})
Hematite (α-Fe_2O_3)	13.20	-17.10	-5×10^{-5}
Manceau Model (FeOOH)	10.58	-17.10	-5×10^{-5}
Michel Model (Fe_5O_8H)	12.10	-17.10	-5×10^{-5}
Goethite (α-FeOOH)	11.02	-17.10	-5×10^{-5}
Lepidocrocite (γ-FeOOH)	10.26	-17.10	-5×10^{-5}
Orthorhombic (Fe_5O_8H)	12.47	-17.10	-5×10^{-5}

Table S2: Details of the surface entropy for each mineral phase investigated. ρ_{H_2O}, $S_{H_2O}^{Chem}$, and $S_{H_2O}^{Phys}$ are respectively the density of water, the entropy of chemisorbed and physisorbed water. While ρ_{H_2O} has been calculated in this work, the values for $S_{H_2O}^{Chem}$ and $S_{H_2O}^{Phys}$ were obtained from the work of Hiemstra.[1]

For each mineral phase, the temperature-dependence of the surface energy has been calculated using the following generic equation:

$$\gamma(T) = \Delta H_{surf}^{h} - T(\rho_{H_2O} \times S_{H_2O}^{Chem} + S_{H_2O}^{Phys})$$

Table S3 summarizes the values of $\gamma(T)$ for the mineral phases considered.

Mineral phases	Temperature (K)											
	0	100	200	298.15	300	400	500	600	700	800	900	1000
Hematite	0.750	0.778	0.805	0.832	0.833	0.860	0.888	0.915	0.943	0.971	0.998	1.026
Manceau Model[a]	0.400	0.423	0.446	0.469	0.469	0.492	0.515	0.539	0.562	0.585	0.608	0.631
Manceau Model[b]	0.100	0.123	0.146	0.169	0.169	0.192	0.215	0.239	0.262	0.285	0.308	0.331
Michel Model[a]	0.400	0.426	0.451	0.477	0.477	0.503	0.528	0.554	0.580	0.606	0.631	0.657
Michel Model[b]	0.100	0.126	0.151	0.177	0.177	0.203	0.228	0.254	0.280	0.306	0.331	0.357
Goethite	0.550	0.574	0.598	0.621	0.622	0.645	0.669	0.693	0.717	0.741	0.765	0.788
Lepidocrocite	0.440	0.463	0.485	0.507	0.508	0.530	0.553	0.575	0.598	0.620	0.643	0.665
Orthorhombic	0.590	0.616	0.643	0.668	0.669	0.695	0.722	0.748	0.774	0.801	0.827	0.853

Table S3: Calculated values of $\gamma(T)$ in J/m^2 at different temperatures.

[a] used $\Delta H_{surf}^{h} = 0.40$ J/m^2.

[b] used $\Delta H_{surf}^{h} = 0.10$ J/m^2.

- **Effect of different (T,pH_2O) conditions on relative phase stability:**

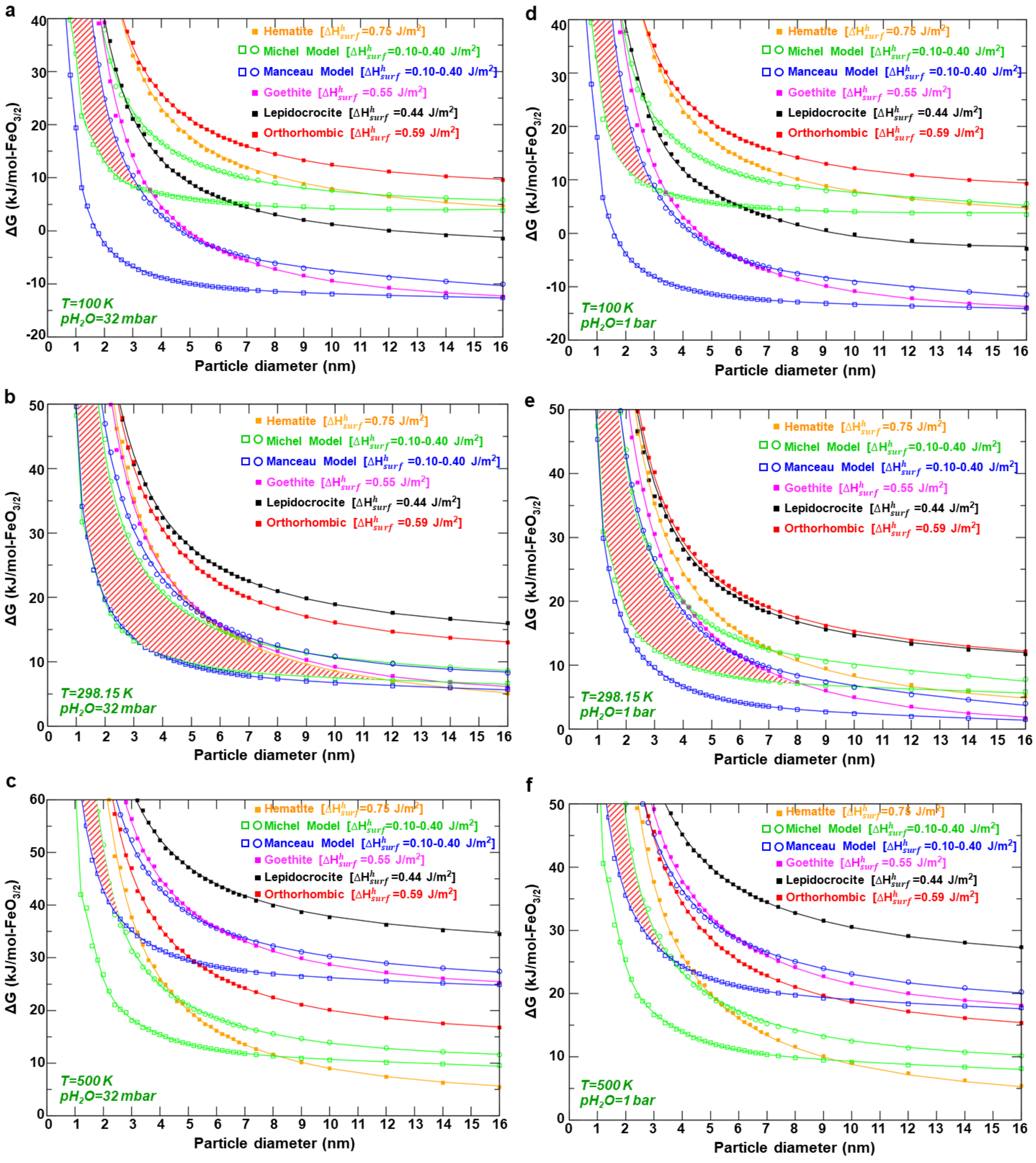

Figure S1: Particle-size dependency of the relative phase stability of the Manceau and Michel ferrihydrite models as well as for goethite, lepidocrocite, hematite and the orthorhombic phase for pH_2O=32 mbar and pH_2O=1 bar, and (a,d) T=100 K, (b,e) T=298.15 K, and (c,f) T=500 K. For the two ferrihydrite models, the curves with open squares were obtained using ΔH^h_{surf}=0.10 J/m^2, while the curves with open circles used ΔH^h_{surf}=0.40 J/m^2. Square filled curves used ΔH^h_{surf} calculated for hydroxylated periodic surface crystals. The red hatched area represents the size-range of particles for which the two ferrihydrite models can potentially coexist.

- **AIT calculated Gibbs free energy and equilibrium reactions:**

Mineral phase	$\Delta G_f^{\text{Calc.}}$ (kJ/mol)	$\Delta G_f^{\text{Expt.}}$ (kJ/mol)	Equilibrium reactions	Log($K_{so}^{\text{Calc.}}$)	Log($K_{so}^{\text{Expt.}}$)
Hematite	-747.375	-744.400[2]	$\mathrm{Fe_2O_3 + 6H^+ \leftrightarrow 2Fe^{3+} + 3H_2O}$	-3.627	-2.101
Michel model (Fe_5O_8H)	-1958.630	n/a	$\mathrm{Fe_5O_8H + 15H^+ \leftrightarrow 5Fe^{3+} + 8H_2O}$	-4.257	n/a
Manceau model (FeOOH)	-486.942	n/a	$\mathrm{FeOOH + 3H^+ \leftrightarrow Fe^{3+} + 2H_2O}$	-1.049	n/a
Goethite	-490.372	-490.600[2]	$\mathrm{FeOOH + 3H^+ \leftrightarrow Fe^{3+} + 2H_2O}$	-1.650	-1.020
Lepidocrocite	-479.810	-482.700[2]	$\mathrm{FeOOH + 3H^+ \leftrightarrow Fe^{3+} + 2H_2O}$	-0.202	0.365
Orthorhombic (Fe_5O_8H)	-1944.192	n/a	$\mathrm{Fe_5O_8H + 15H^+ \leftrightarrow 5Fe^{3+} + 8H_2O}$	-1.725	n/a
H_2O	-235.232	-237.141[3]			
Fe^{3+}	n/a	-10.500[4]			

Table S4: Comparison of AIT calculated and experimental Gibbs free energies and solubilities for virtual bulk lattices of the various mineral phases considered at T=298.15 K and pH$_2$O=32 mbar.

- **Equilibrium solubility and hydrolysis reactions:**

Based on the equilibrium reactions shown in Table S4, the equilibrium solubility of [Fe(III)] for each mineral phase has been calculated by following the methodology given in Ref[4,5] by:

$$K_{\mathrm{Fe_2O_3}} = \frac{a_{\mathrm{Fe}}^2 a_{\mathrm{H_2O}}^3}{a_{\mathrm{H}}^6}$$

$$K_{\mathrm{Fe_5O_8H}} = \frac{a_{\mathrm{Fe}}^5 a_{\mathrm{H_2O}}^8}{a_{\mathrm{H}}^{15}}$$

$$K_{\mathrm{FeOOH}} = \frac{a_{\mathrm{Fe}} a_{\mathrm{H_2O}}^2}{a_{\mathrm{H}}^3}$$

Where a_i is the activity of the species i and is defined by the product between the activity coefficient (γ_i) and the concentration of the species i such that $a_i = \gamma_i \times [i]$. Considering pure water as the medium for the chemical reactions, we used the Davies formula to calculate the activity coefficients of the charged species involved in the chemical reactions, as well as the activity of water. The use of the Davies formula is justified for solutions with ionic strength lower than 0.3 mol/kg.

$$\gamma = 10^{-a z_i^2\left(\frac{\sqrt{I}}{1+\sqrt{I}} - 0.3I\right)},$$

where a is the Debye-Hückel parameter having the value of 0.50948, z_i is the charge of the species, and I (in mol/kg) is the ionic strength of the solution. The water activity has been determined by the following equation:

$$(H_2O) = \frac{[H_2O]}{[H_2O]+\sum_i[\text{solute}]},$$

where $[H_2O]$=55.5099 mol/kg.

The hydrolysis equilibrium reactions[4,5] considered in the calculations are:

$$Fe^{3+} + H_2O \leftrightarrow Fe(OH)^{2+} + H^+$$

$$Fe^{3+} + 2H_2O \leftrightarrow Fe(OH)_2^+ + 2H^+$$

$$Fe^{3+} + 3H_2O \leftrightarrow Fe(OH)_3^0 + 3H^+$$

$$Fe^{3+} + 4H_2O \leftrightarrow Fe(OH)_4^- + 4H^+$$

- **Effect of pH on the solubility of [Fe(III)] of iron-(oxyhydr)oxide minerals:**

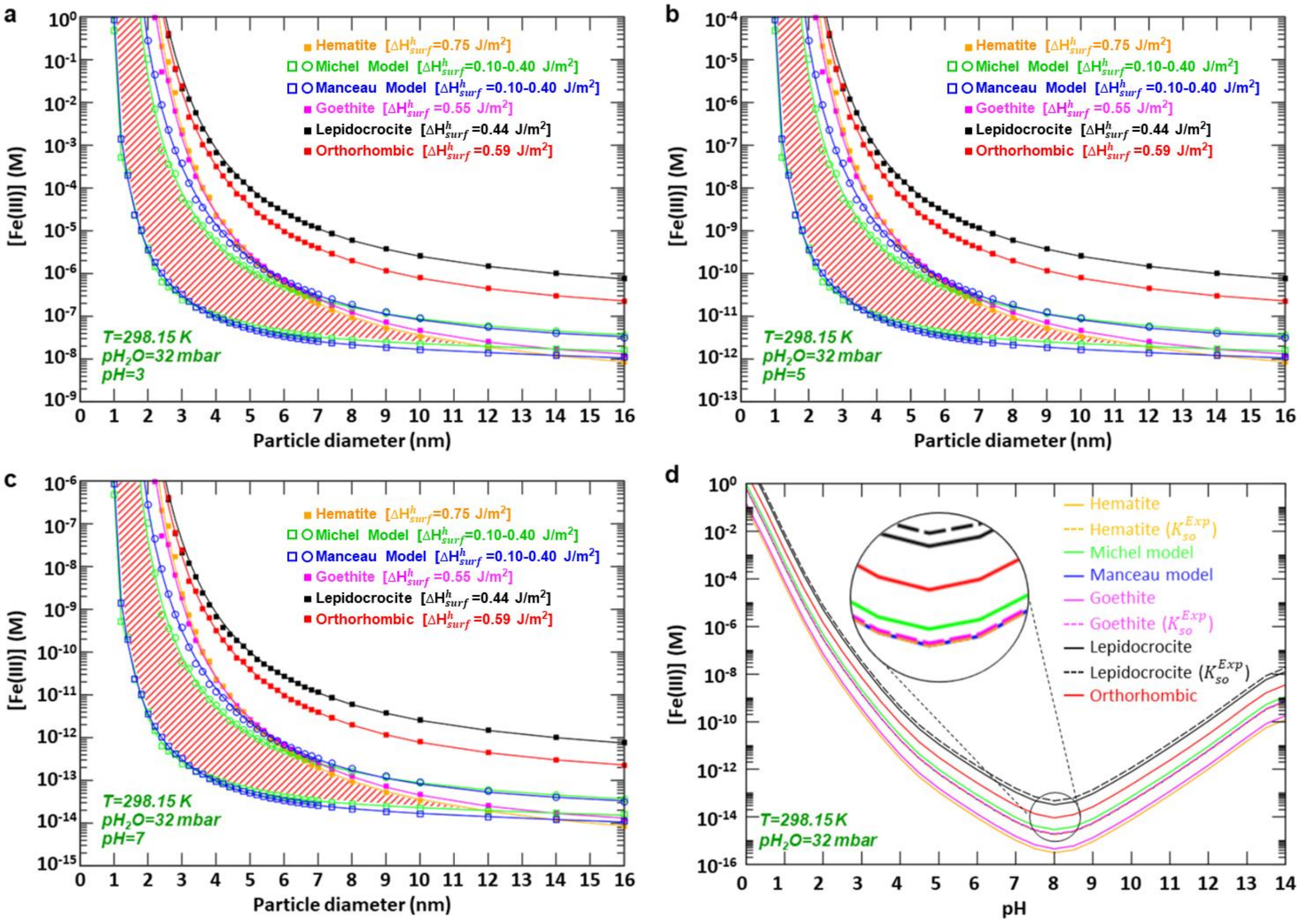

Figure S2: Equilibrium solubility of [Fe(III)] for the various minerals in pure water. (a-c) Particle-size dependence of [Fe(III)] solubility at pH=3, 5, and 7. The red hatched area represents the size-range of particles for which the two ferrihydrite models can potentially coexist. (d) pH-dependence of [Fe(III)] solubility of iron (oxyhydr)oxide phases considered as virtual bulk lattices, with Gibbs free energy calculated from AIT and compared to experimental values.

- **Supplementary References:**

1. Hiemstra, T. Formation, stability, and solubility of metal oxide nanoparticles: Surface entropy, enthalpy, and free energy of ferrihydrite. *Geochim. Cosmochim. Acta* **158**, 179-198 (2015).
2. Navrotsky, A.; Mazeina, L.; Majzlan, J. Size-driven structural and thermodynamic complexity in iron oxides. *Science* **319**, 1635-1638 (2008).
3. *NIST-JANAF Thermochemical Tables*, 4th ed., edited by J. M. W. Chase American Chemical Society, Washington, DC, **1998**.
4. Millero, F. J; Yao, W.; Aicher, J. The separation of Fe(II) and Fe(III) in natural waters. *Mar. Chem.* **50**, 21-39 (1995).
5. Liu, X.; Millero, F. J. The solubility of iron hydroxide in sodium chloride solutions. *Geochim. Cosmochim. Acta* **63**, 3487-3497 (1999).
